# Successful treatment of out-of-hospital cardiac arrest is still based on quick activation of the chain of survival

**DOI:** 10.3389/fpubh.2023.1126503

**Published:** 2023-04-11

**Authors:** Laura Borgstedt, Stefan J. Schaller, Daniel Goudkamp, Kristina Fuest, Bernhard Ulm, Bettina Jungwirth, Manfred Blobner, Sebastian Schmid

**Affiliations:** ^1^Department of Anesthesiology and Intensive Care Medicine, School of Medicine, Technical University of Munich, Munich, Germany; ^2^Department of Anesthesiology and Operative Intensive Care Medicine (CVK, CCM), Charité - Universitaetsmedizin Berlin, Berlin, Germany; ^3^Department of Anesthesiology and Intensive Care, Faculty of Medicine, University of Ulm, Ulm, Germany

**Keywords:** out-of-hospital cardiac arrest, cardiopulmonary resuscitation, defibrillation, bystander, return of spontaneous circulation, initial heart rhythm, Germany, public space

## Abstract

**Background and goal of study:**

Cardiopulmonary resuscitation (CPR) in prehospital care is a major reason for emergency medical service (EMS) dispatches. CPR outcome depends on various factors, such as bystander CPR and initial heart rhythm. Our aim was to investigate whether short-term outcomes such as the return of spontaneous circulation (ROSC) and hospital admission with spontaneous circulation differ depending on the location of the out-of-hospital cardiac arrest (OHCA). In addition, we assessed further aspects of CPR performance.

**Materials and methods:**

In this monocentric retrospective study, protocols of a prehospital physician-staffed EMS located in Munich, Germany, were evaluated using the Mann–Whitney U-test, chi-square test, and a multifactor logistic regression model.

**Results and discussion:**

Of the 12,073 cases between 1 January 2014 and 31 December 2017, 723 EMS responses with OHCA were analyzed. In 393 of these cases, CPR was performed. The incidence of ROSC did not differ between public and non-public spaces (*p* = 0.4), but patients with OHCA in public spaces were more often admitted to the hospital with spontaneous circulation (*p* = 0.011). Shockable initial rhythm was not different between locations (*p* = 0.2), but defibrillation was performed significantly more often in public places (*p* < 0.001). Multivariate analyses showed that hospital admission with spontaneous circulation was more likely in patients with shockable initial heart rhythm (*p* < 0.001) and if CPR was started by an emergency physician (*p* = 0.006).

**Conclusion:**

The location of OHCA did not seem to affect the incidence of ROSC, although patients in public spaces had a higher chance to be admitted to the hospital with spontaneous circulation. Shockable initial heart rhythm, defibrillation, and the start of resuscitative efforts by an emergency physician were associated with higher chances of hospital admission with spontaneous circulation. Bystander CPR and bystander use of automated external defibrillators were low overall, emphasizing the importance of bystander education and training in order to enhance the chain of survival.

## 1. Introduction

With approximately 51,970 cases in 2019 (1), out-of-hospital cardiac arrest (OHCA) is still a major public health issue in Germany. Although outcomes have improved over the last decade (2), overall survival to hospital discharge and overall survival with favorable neurological outcomes are still reported at approximately 10% (1, 3, 4). Survival rates in men are 7.9%, whereas survival rates in women are reported at 3.7% (2).

Previous studies suggested that early defibrillation and bystander cardiopulmonary resuscitation (CPR) improve survival after OHCA (5–8). This has led to several efforts to improve life-sustaining measures prior to the arrival of the emergency medical service (EMS): automated external defibrillator dissemination, public campaigns to increase awareness for cardiac arrest and to distribute CPR education, and telephone CPR (T-CPR), also known as dispatch-assisted CPR.

Telephone CPR was first integrated into the 2010 guidelines for resuscitation by the European Resuscitation Council, where further development of AED programs was also encouraged (9). Following these recommendations, Bavaria was the first federal state in Germany to implement telephone CPR in its emergency medical dispatch protocols in 2013 (10).

The organization of the EMS in Germany follows regulations by the local government of each of the 16 federal states but is often delegated to the district level. It is a physician-based system in which usually an emergency ambulance (staffed with at least two paramedics) and the physician-staffed EMS are not co-located, i.e., the rendezvous system.

If the reported emergency involves potentially life-threatening, cardiovascular, or neurological conditions (for example, OHCA, myocardial infarction, or unconsciousness), dyspnea, severe intoxication, severe trauma, pediatric patients, mass-casualty incidents, or if the need for analgesics or anesthesia seems very likely, the emergency physician is deployed automatically by the call handler at the emergency medical dispatch center. Alternatively, the emergency physician can be radioed in for support by the paramedics at the scene (11). On the one hand, the rendezvous system allows the emergency physician to be available for other emergencies quicker if the patient can be handled and admitted to the hospital only by paramedics. On the other hand, the paramedics might need to perform CPR and handle a critically ill or injured patient without support until the emergency physician arrives.

To qualify as an emergency physician in Germany, physicians must complete an 80-h course in prehospital emergency medicine, 2 years of clinical training with at least 6 months in anesthesia or intensive care, participate in 50 runs with a certified emergency physician, and pass a board examination. By law, only emergency physicians may perform invasive medical procedures on a patient and pronounce a patient dead. Paramedics undergo 3 years of full-time, on-the-job training with theoretical basics, practical training, and work assignments in the EMS. The medical procedures that a paramedic is allowed to perform are limited and regulated by law. However, in life-threatening cases such as resuscitation, he or she can act like an emergency doctor, with the exception of determining death.

Despite efforts to improve the chain of survival in OHCA (e.g., increasing public awareness for OHCA, education in basic life support, AED dissemination, and many others), the return of spontaneous circulation (ROSC) is approximately 33% (1), while survival to hospital discharge is described at approximately 10% in the Western world (1, 12, 13). This study aimed to analyze factors contributing to ROSC and hospital admission with spontaneous circulation after an OHCA. The main focus here was the location of the OHCA (public space versus non-public space). Further factors were the initial arrest rhythm and the person who initiated CPR. In addition, aspects of resuscitation performance such as delay in initiation, bystander CPR, use of AEDs, defibrillation, use of automated mechanical chest compression devices (AMCCD), and transportation under CPR were examined.

## 2. Methods

After the approval of the ethics committee of the TUM School of Medicine (508/16 S-S), this monocentric retrospective study evaluated protocols of dispatches from a prehospital physician-staffed EMS. The ethics committee waived the requirement for informed consent, and the study was carried out in accordance with the Declaration of Helsinki.

The ground-based EMS is located at Fire Department 10 in Munich-Riem (Joseph-Wild-Str. 15, 81,829 Munich, Germany). It consists of a specially equipped vehicle staffed with a firefighter (who is also a trained paramedic) and a physician (specialized in either anesthesiology, trauma, or general surgery) working at a university hospital (Klinikum rechts der Isar, Technical University Munich, Munich, Germany). The firefighter also serves as a driver. The EMS is equipped according to DIN 75079 with an ECG, defibrillator, external pacemaker, ventilator, suction device, and medication and is not fit for patient transportation.

### 2.1. Data collection

Data were collected from standardized EMS forms (DIVI version 4.2, DIVI version 5.0, and DIVI version 5.1) (refer to Supplementary Figures 1–3) containing patient and field data, as described before (14). Data were stored and analyzed in an anonymized fashion. OHCA was defined as all persons (age >18 years) reported as unresponsive, apneic or without normal breathing, and/or without a palpable pulse who received basic life support and/or advanced life support as defined in the resuscitation guidelines (15, 16).

All cases with OHCA were assessed regarding sex, age, location (public space/non-public space), initial heart rhythm, witnessed/unwitnessed OHCA, bystander use of AED, T-CPR (yes/no), initiation of CPR (bystander/emergency service/emergency physician), defibrillation by EMS, use of an AMCCD, delay, suspected underlying cause of OHCA/patient history, ROSC, and hospital admission (yes/no). Delay was defined as a non-witnessed OHCA or initiation of CPR by EMS or an emergency physician. Data were also stratified by the location in which OHCA occurred. Non-public space included every emergency in a private or retirement home. Public space was defined as every emergency that did not happen in one of the aforementioned locations. The primary endpoint of this study was hospital admission with spontaneous circulation. We further assessed documentation quality regarding missing parameters and did a confirmatory analysis with complete CPR datasets only.

### 2.2. Statistical analysis

The results are presented as median [interquartile range (IQR)] or absolute and relative frequencies. For data points with adequate documentation frequency (>90%), univariate analysis was performed using Mann–Whitney U-test or chi-square test as appropriate. In the case of significance, the variable was included in multivariate analysis [refer also to (14)]. In order to correct for confounding factors, all variables that showed a univariate value of *p* of ≤0.2 were included in the multivariate regression model. Multivariate analyses were performed with binary logistic regression models. Odds ratios are reported with a 95% confidence interval. As a secondary analysis, classification trees using the CART method were performed with the primary endpoint of hospital admission with spontaneous circulation. The parameters of the classification tree were a minimum number of 30 patients per node to be further split and a minimum number of 20 patients per end node. A significance level of 0.05 was defined.

Calculations were done with R version 3.6.3 (R Foundation for Statistical Computing, Vienna, Austria).

## 3. Results

**Figure 1:**
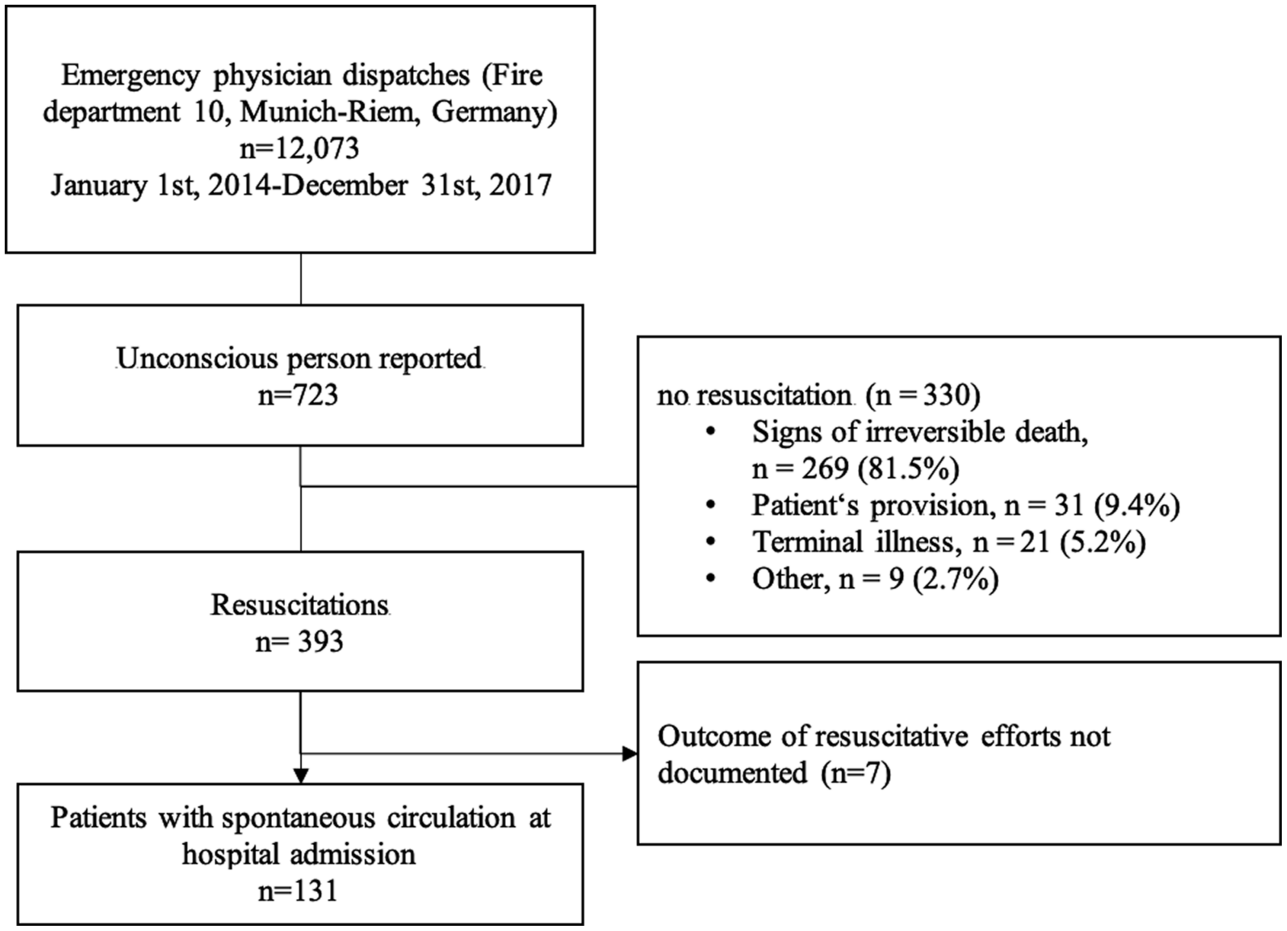
CONSORT flow diagram.

Between 1 January 2014 and 31 December 2017, the emergency physician vehicle of Fire Department 10 in Munich-Riem, Germany, handled 12,073 cases (further described in Supplementary Table 1). Of those, 723 cases involved OHCA, and in 393 cases, CPR was performed. CPR was not initiated if the patient showed signs of irreversible death (*n* = 269, 81.5%), if a living will or other advance directive including a “do not resuscitate” order was presented (*n* = 31, 9.4%), if the patients were terminally ill (n = 21, 5.2%), or for other unspecified reasons (*n* = 9, 2.7%) (Figure 1).

The 393 CPR cases are further described in Table 1. In 336 cases, a complete dataset was available. Following CPR, ROSC was achieved at least once in 190 patients (48.3%). 166 patients (42.2%) were admitted to the hospital with spontaneous circulation, and 87 patients (22.1%) were admitted with ongoing resuscitation (Table 2). Variables associated with ROSC were shockable initial heart rhythm (*p* < 0.001) and if defibrillation had been performed during resuscitation (*p* < 0.001). ROSC was more often achieved if resuscitation had been started by an emergency physician (*p* = 0.003). If resuscitative efforts had been started with a delay, ROSC occurred significantly less (*p* = 0.002) (Table 3). The confirmatory analysis of complete datasets (*n* = 336) also showed a significance for shockable initial heart rhythm (*p* < 0.001), defibrillation (*p* < 0.001), start of CPR by emergency physicians (*p* = 0.001), and delay (*p* = 0.004), and supports the findings of the primary analysis (Table 3).

**Table 1:** Depiction of cardiopulmonary resuscitation (CPR) cases.

	All CPRs (*n* = 393)	CPRs with full dataset (*n* = 336)
Sex		
Female	147 (37.4)	127 (37.8)
Male	246 (62.6)	209 (62.2)
Age; median [IQR]	74 [61, 83]	74 [61, 83]
Unknown (n)	7	
Age split		
<60	93 (24.1)	80 (23.8)
60–79	168 (43.5)	144 (42.9)
≥80	125 (32.4)	112 (33.3)
Unknown (*n*)	7	
Location		
Non-public space	285 (72.5)	249 (74.1)
Public space	108 (27.5)	87 (25.9)
Suspected underlying cause		
Abdominal	4 (1.1)	3 (1.0)
Respiratory	10 (2.8)	9 (2.9)
Cardiovascular	288 (80.0)	256 (82.1)
Pediatric	1 (0.3)	1 (0.3)
Other	47 (13.1)	34 (10.9)
Central nervous system	10 (2.8)	9 (2.9)
Unknown (n)	33	34

Characteristics of all CPR cases and all CPR cases with full dataset, including the location of out-of-hospital cardiac arrest. All variables are presented as numbers (*n*) with frequencies (%). Variables presented with median and interquartile range [IQR] are marked accordingly.

**Table 2:** Patients and cardiopulmonary resuscitation (CPR) characteristics stratified by location of out-of-hospital cardiac arrest (OHCA).

	Non-public space (*n* = 285)	Public space (*n* = 108)	*p*-value	Missing (%)
Sex				
Female	121 (42.5)	26 (24.1)	0.001	0.0
Male	164 (57.5)	82 (75.9)		
Age; median [IQR]	77 [67, 85]	64 [51, 75]	< 0.001	1.8
Age split				
<60	47 (16.5)	46 (45.1)	< 0.001	1.8
60–79	124 (43.7)	44 (43.1)		
≥80	113 (39.8)	12 (11.8)		
Bystander CPR				
Yes	77 (27.6)	40 (38.1)	0.062	2.3
No	202 (72.4)	65 (61.9)		
ROSC				
Yes	134 (47.0)	56 (51.9)	0.457	0.0
No	151 (53.0)	52 (48.1)		
CPR successful				
Yes	109 (38.8)	57 (53.8)	0.011	1.5
No	172 (61.2)	49 (46.2)		
Ongoing CPR hospital admission				
Yes	49 (19.5)	38 (40.4)	< 0.001	12.2
No	202 (80.5)	56 (59.6)		
AMCCD				
Yes	81 (30.5)	47 (46.1)	0.007	6.4
No	119 (66.5)	121 (64.0)		
Defibrillation				
Yes	95 (35.6)	60 (58.3)	< 0.001	5.9
No	172 (64.4)	43 (41.7)		
Bystander AED				
Yes	9 (3.5)	4 (4.0)	1.000	8.4
No	251 (96.5)	96 (96.0)		
Start CPR				
Emergency physician	63 (25.2)	25 (26.9)	0.106	12.7
Emergency service	91 (36.4)	23 (24.7)		
Bystander	96 (38.4)	45 (48.4)		
Delay				
Yes	123 (56.2)	34 (38.2)	0.006	21.6
No	96 (43.8)	55 (61.8)		

All variables are presented as numbers (*n*) with frequencies (%). Variables presented with median and interquartile range [IQR] are marked accordingly. ROSC, Return of spontaneous circulation; AMCCD, Automated mechanical chest compression device; AED, Automated external defibrillator; CPR successful, Hospital admission with spontaneous circulation; Delay, non-witnessed OHCA or the initiation of CPR by EMS or emergency physician.

**Table 3:** Univariate analysis of the return of spontaneous circulation (ROSC) at least once compared to no ROSC.

ROSC	All resuscitations			Resuscitations with full dataset		
	Yes (*n* = 190)	No (*n* = 203)	*p*-value	Yes (*n* = 165)	No (*n* = 171)	*p*-value
Sex			0.5			0.4
Female	68 (35.8)	79 (38.9)		59 (35.8)	68 (39.8)	
Male	122 (64.2)	124 (61.1)		106 (64.2)	103 (60.2)	
Age; median [IQR]	72 [60, 81]	74 [64, 84]	0.2	73 [61, 83]	75 [63, 84]	0.5
Age split			0.6			> 0.9
<60	48 (25.5)	45 (22.7)		40 (24.2)	40 (23.4)	
60–79	84 (44.7)	84 (42.4)		71 (43.0)	73 (42.7)	
≥80	56 (29.8)	69 (34.8)		54 (32.7)	58 (33.9)	
Bystander CPR			0.9			0.3
Yes	57 (30.8)	60 (30.2)		44 (26.7)	54 (31.6)	
No	128 (69.2)	139 (69.8)		121 (73.3)	117 (68.4)	
AMCCD			0.6			0.8
Yes	60 (33.5)	68 (36.0)		52 (31.5)	55 (32.7)	
No	119 (66.5)	121 (64.0)		113 (68.5)	113 (67.3)	
Defibrillation			< 0.001			< 0.001
Yes	98 (54.1)	57 (30.2)		85 (51.5)	49 (28.7)	
No	83 (45.9)	132 (69.8)		80 (48.5)	122 (71.3)	
Bystander AED			0.3			0.3
Yes	8 (4.6)	5 (2.7)		7 (4.2)	4 (2.3)	
No	166 (95.4)	181 (97.3)		158 (95.8)	167 (97.7)	
Start CPR			0.003			< 0.001
Emergency physician	56 (33.5)	32 (18.2)		56 (33.9)	29 (17.0)	
Emergency service	45 (26.9)	69 (39.2)		45 (27.3)	69 (40.4)	
Bystander	66 (39.5)	75 (42.6)		64 (38.8)	73 (42.7)	
Telephone CPR			> 0.9			0.7
Yes	13 (7.5)	14 (7.4)		10 (6.1)	12 (7.0)	
No	161 (92.5)	174 (92.6)		154 (93.9)	159 (93.0)	
Initial heart rhythm			< 0.001			< 0.001
Shockable	53 (27.9)	21 (10.3)		44 (26.7)	20 (11.7)	
Non-shockable	137 (72.1)	182 (89.7)		121 (73.3)	151 (88.3)	
Delay			0.002			0.004
Yes	66 (42.3)	91 (59.9)		64 (43.2)	84 (60.4)	
No	90 (57.7)	61 (40.1)		84 (56.8)	55 (39.6)	
Location			0.4			0.3
Non-public space	134 (70.5)	151 (74.4)		118 (71.5)	131 (76.6)	
Public space	56 (29.5)	52 (25.6)		47 (28.5)	40 (23.4)	
Defibrillation & heart rhythm			< 0.001			< 0.001
Defibrillation yes, rhythm shockable	44 (24.3)	19 (10.1)		36 (21.8)	18 (10.5)	
Defibrillation yes, non-shockable rhythm	54 (29.8)	38 (20.1)		49 (29.7)	31 (18.1)	
Defibrillation no, shockable rhythm	8 (4.4)	2 (1.1)		8 (4.8)	2 (1.2)	
Defibrillation no, non-shockable rhythm	75 (41.4)	130 (68.8)		72 (43.6)	120 (70.2)	

All variables are presented as numbers (*n*) with frequencies (%). Variables presented with median and interquartile range [IQR] are marked accordingly. CPR, Cardiopulmonary resuscitation; AMCCD, Automated mechanical chest compression device; AED, Automated external defibrillator; Delay, Non-witnessed OHCA or the initiation of CPR by EMS or emergency physician.

Stratification by location of OHCA showed that in public spaces, younger patients and more male patients were encountered. In addition, initiation of CPR was more often delayed in non-public spaces (Age: *p* < 0.001; sex: *p* < 0.001; delay before initiation of CPR: *p* = 0.006; Table 2). Resuscitative efforts leading to ROSC at least once did not differ between locations (non-public space 47.0% vs. public space 51.9%, *p* = 0.46). Concerning transportation and hospital admission, more patients with OHCA in public spaces were admitted to the hospital with ROSC and were subjected to intra-arrest transportation (Table 2). In public spaces, automated mechanical chest compression devices (AMCCDs) were used, and defibrillation was performed more often (AMCCDs: *p* = 0.007; defibrillation: *p* < 0.001, Table 2). No difference in initial heart rhythm between locations was found (*p* = 0.23). The use of bystander automated external defibrillators (AEDs) was reported to be 3.5% in non-public spaces and 4.0% in public spaces (*p* = 1.0). Telephone CPR was performed in 7.6% of non-public spaces and 7.1% of public spaces (*p* = 1.0).

If CPR was started by the emergency service, multivariate logistic regression showed an odds ratio of 0.34 for admission to the hospital with ROSC and an odds ratio (OR) of 0.45 in the case of a bystander. Non-shockable initial heart rhythm was attributed with an OR of 0.23 for admission to the hospital with ROSC. Logistic regression showed an OR of 0.13 for admission to a hospital with ROSC in a public space if CPR was started by emergency services. An OR of 0.10 for admission to a hospital with ROSC was found for non-shockable initial heart rhythm in a public space. Logistic regression did not show significant differences in OR for admission to a hospital with ROSC if a delay was suspected, irrespective of location (Table 4).

**Table 4:** Multivariate logistic regression models for the outcome of cardiopulmonary resuscitation (CPR), defined by hospital admission with spontaneous circulation.

	All patients (*n* = 336)		Non-public space (*n* = 249)		Public space (*n* = 87)	
	OR [95% CI]	*p*-value	OR [95% CI]	*p*-value	OR [95% CI]	*p*-value
Start CPR		0.006		0.11		0.022
Emergency physician^#^	-		-		-	
Emergency service	0.34 [0.17, 0.68]	0.002	0.44 [0.20, 0.95]	0.038	0.13 [0.02, 0.65]	0.018
Bystander	0.45 [0.24, 0.85]	0.015	0.58 [0.27, 1.22]	0.2	0.21 [0.05, 0.76]	0.024
Age split		0.6		0.7		0.7
< 60^#^	-		-		-	
60–79	1.35 [0.70, 2.63]	0.4	1.32 [0.57, 3.11]	0.5	1.49 [0.47, 5.00]	0.5
≥80	1.39 [0.67, 2.91]	0.4	1.48 [0.63, 3.57]	0.4	0.91 [0.16, 5.35]	>0.9
Location		0.8				
Non-public space^#^	-		-		-	
Public space	1.10 [0.60, 2.03]	0.8				
Defibrillation & heart rhythm		<0.001		<0.001		0.011
Defibrillation yes, shockable rhythm^#^	-		-		-	
Defibrillation yes, non-shockable rhythm	0.72 [0.31, 1.64]	0.4	1.18 [0.42, 3.28]	0.8	0.23 [0.04, 1.03]	0.071
Defibrillation no, shockable rhythm	0.75 [0.14, 5.74]	0.7	0.72 [0.12, 5.99]	0.7	1,288,806 [0.00, NA]	>0.9
Defibrillation no, non-shockable rhythm	0.23 [0.11, 0.47]	<0.001	0.30 [0.12, 0.68]	0.005	0.10 [0.02, 0.45]	0.005
Delay		0.081		0.2		0.3
Yes^#^	-		-		-	
No	1.60 [0.94, 2.72]	0.081	1.55 [0.85, 2.86]	0.2	1.79 [0.55, 5.97]	0.3

Three binary regression models were calculated: first a model for all CPRs with full datasets and additionally one model for each of the subgroups public spaces and non-public spaces. Variables are given as OR (odds ratio) and 95% confidence interval (CI) and are referenced by the variables marked with ^#^. Delay, Non-witnessed OHCA or the initiation of CPR by EMS or emergency physician.

**Figure 2:**
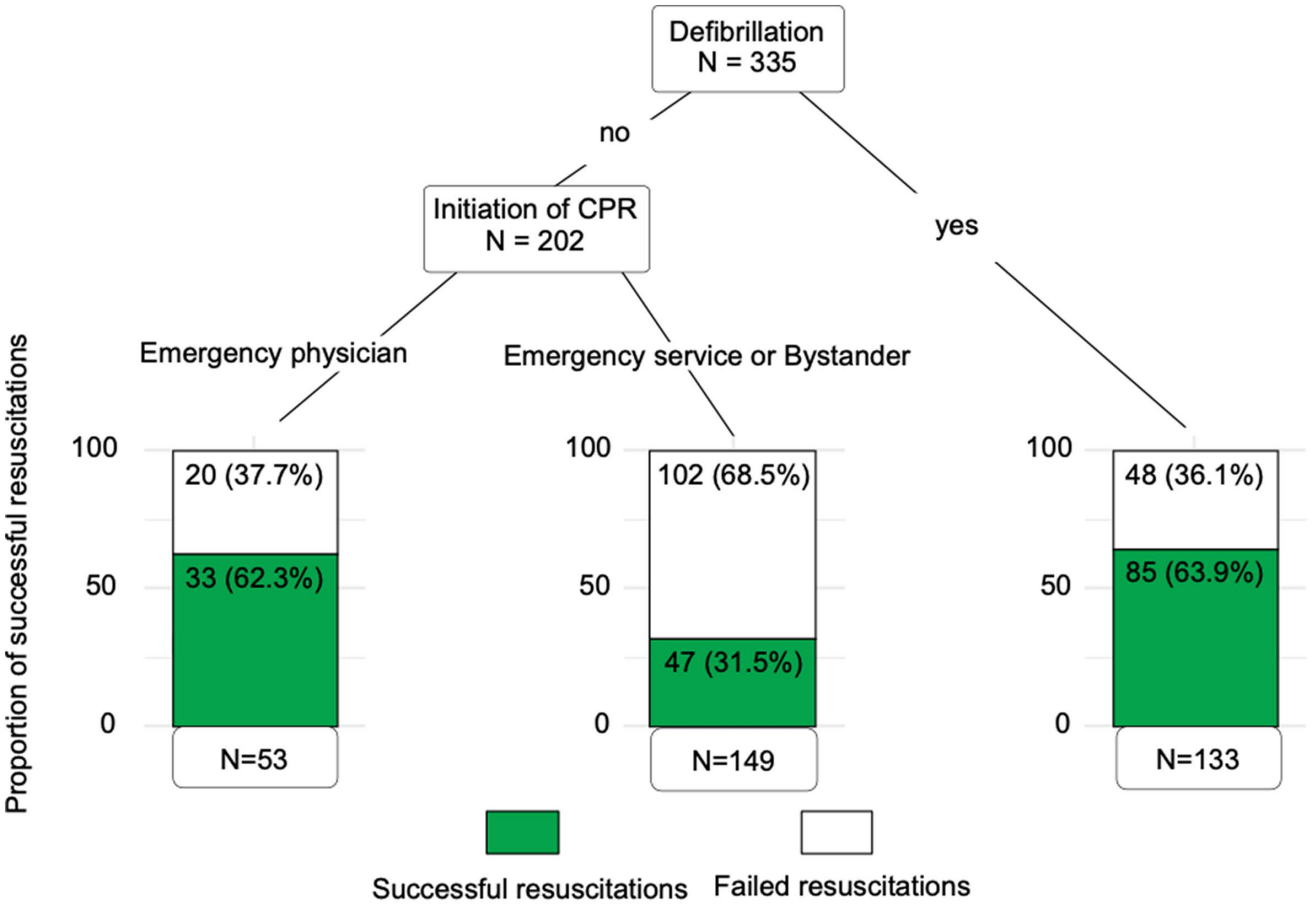
Classification tree. Classification tree depicting the outcome of resuscitations. Independent variables of the classification tree were defibrillation, initiation of CPR, initial rhythm, age, location of the OHCA, and delay. The parameters of the classification tree were a minimum number of 30 patients per node to be further split and a minimum number of 20 patients per end node. Defibrillation was found as the first node, and if defibrillation was not performed, a second node was found in the group that started resuscitative efforts. The highest chances of successful resuscitations were found in patients where defibrillation was performed. The lowest chances of successful resuscitations were found in 149 patients where defibrillation was not performed and where CPR was started by emergency services or bystanders.

With the initial split at defibrillation (yes/no), CART analysis showed that if defibrillation was performed, the rate of successful CPR was 63.4%. If defibrillation was not indicated and CPR was begun by an emergency physician, the rate of successful CPR was 62.3%. If CPR in a patient with a non-shockable initial heart rhythm was started by emergency services or a bystander, the rate of success was 31.5% (Figure 2).

## 4. Discussion

A total of 393 cases of CPR following OHCA were retrospectively assessed. Regarding the location of OHCA, the incidence of ROSC did not differ, although more patients in public spaces survived to hospital admission. Defibrillation was performed more often in patients with cardiac arrest in public spaces, and they were also more often transferred to the hospital with ongoing CPR. In addition, patients encountered in public spaces were younger and more often male. Factors associated with higher chances of survival to hospital admission were shockable initial heart rhythm, defibrillation, and start of resuscitative efforts by emergency physicians. AMCCDs were also used more often in patients encountered in public spaces. A delay in the initiation of CPR seemed more common in non-public spaces.

We did not find significant differences in bystander CPR, ROSC at least once, or use of bystander AED if data were stratified by location. Bystander CPR (approximately 30% of all OHCA cases) and bystander use of AED (approximately 4% of all OHCA cases) were overall low. A recent study from Japan found even lower rates of bystander CPR and bystander use of an AED with 20.8 and 2.0%, respectively (17). USA-based studies assessing larger cohorts found rates of 40% (18) to 49% (19) for bystander CPR and 8.4% for bystander AED use (20). To the best of our knowledge, there are only a few studies investigating bystander CPR and bystander use of AED in OHCA in Germany. Recently, Bohm et al. investigated sports-related sudden cardiac arrests in young patients in Germany and France and found rates of bystander CPR of 82.6% and bystander AED of 7.5% (21). Given the facts that mortality rates are lowest when an AED is placed within 3–5 min of a cardiac arrest (22) and that OHCA outcomes are poorer in Germany than in other countries (12), our study too shows the need for more awareness and education in OHCA as well as basic life support and bystander use of AEDs combined with an increase in public accessibility (23). This is in part supported by our finding that patients had higher chances of ROSC when resuscitative efforts were begun by emergency physicians. We believe that this result might be influenced by the fact that the emergency physician is the only person in Germany allowed to decide whether CPR is not begun or stopped. The emergency service is obliged to start resuscitative efforts in an unresponsive, apneic person in the absence of injuries incompatible with life (24). In contrast, the emergency physician can decide against the initiation of CPR.

In our study, the incidence of ROSC did not differ in non-public compared to public spaces, whereas more patients after OHCA in public spaces received defibrillation and were admitted to hospital with spontaneous circulation. This result is in line with current literature (25) and might be explained by several other findings: Patients with OHCA in public spaces were significantly younger, and more patients were male. Recent studies also identified younger age (25, 26) and being male (27, 28) as favorable factors in OHCA. Whether the latter is due to therapeutic bias or sex-specific differences in pathophysiology remains to be elucidated. In addition, defibrillation was performed more often in public spaces and is known to increase the chances of ROSC, sustained spontaneous circulation, and long-term outcomes after OHCA (29). Shockable rhythm is often early in the OHCA process (30). (Early) defibrillation is not only the recommended treatment but also implies early use of AED or defibrillator and, thus, a reduced chain of survival time. Our study also showed that defibrillation *per se* increased chances of ROSC and might be advisable in CPR even in the absence of a shockable rhythm. As our investigation was of the monocentric and retrospective design, further prospective trials are warranted.

Significantly more patients were transported to the hospital with ongoing CPR after OHCA in public spaces compared to non-public spaces. Whether or not to transfer a patient during resuscitative efforts has long been under debate, and to date, strategies for the transport of these patients differ markedly (31). The European Resuscitation Council Guidelines 2021 in adult advanced life support state that adult patients with non-traumatic OHCA should be considered for transport to a cardiac arrest center according to local protocols (16). An observational study in 2020 found that intra-arrest transport to hospital was associated with a lower probability of hospital discharge compared to on-scene resuscitation (19). Some of the patients for whom resuscitation was initiated in a public space may have been transported to hospital under continued resuscitation, since declaring a patient dead “on the street” involves significant administrative work. With extracorporeal cardiopulmonary resuscitation (ECPR) gaining more and more importance as a rescue method in refractory OHCA (32), the question of how and when to transport a patient with OHCA will still be the subject of research in the years to come.

Our study has several limitations. The data of only one emergency service location in Munich staffed by physicians of one university hospital were collected, as is reflected in the number of cases assessed. In addition, all cases of OHCA were assessed, and there was no differentiation between traumatic or non-traumatic cardiac arrest. Due to the retrospective design and paper documentation, study findings are limited by potential confounding. As data collection stopped at hospital admission, we did not follow-up on mortality or other outcome parameters. In our study, the most relevant documentation on CPR was provided by the emergency physicians. However, the standardized EMS form (see Supplementary material) usually was not filled out in its entirety (19, 33).

## 5. Conclusion

In this study, we found that the location of OHCA did not influence the incidence of ROSC, although patients in public spaces had a higher chance of surviving to hospital admission after ROSC. Furthermore, hospital admission with spontaneous circulation after OHCA was more likely if the first arrest rhythm was shockable, if defibrillation was performed, and if CPR was initiated by the emergency physician. Bystander CPR and bystander use of AED were overall low, emphasizing the importance of bystander education and training in order to strengthen the chain of survival.

## Data availability statement

The raw data supporting the conclusions of this article will be made available by the authors, without undue reservation.

## Ethics statement

The studies involving human participants were reviewed and approved by Ethics committee of the TUM School of Medicine (508/16 S-S). Written informed consent for participation was not required for this study in accordance with the national legislation and the institutional requirements.

## Author contributions

LB: investigation, data curation, formal analysis, validation, writing—original draft, and writing—reviewing and editing. SJS: conceptualization, methodology, investigation, formal analysis, validation, and writing—reviewing and editing. DG: investigation and data acquisition. KF: formal analysis and validation. BU: methodology, data curation, formal analysis, and validation. BJ: supervision and resources. MB: methodology, resources, formal analysis, supervision, and writing—reviewing and editing. SS: methodology, investigation, formal analysis, validation, supervision, writing—original draft, and writing—reviewing and editing. All authors contributed to the article and approved the submitted version.

## Funding

This study received institutional funding by Technical University Munich.

## Conflict of interest

SJS received grants and non-financial support from Reactive Robotics GmbH (Munich, Germany), ASP GmbH (Attendorn, Germany), STIMIT AG (Biel, Switzerland), ESICM (Geneva, Switzerland), grants, personal fees and non-financial support from Fresenius Kabi Deutschland GmbH (Bad Homburg, Germany), grants from the Innovationsfond of The Federal Joint Committee (G-BA), personal fees from Springer Verlag GmbH (Vienna, Austria) for educational purposes and Advanz Pharma GmbH (Bielefeld, Germany), non-financial support from national and international societies (and their congress organizers) in the field of anesthesiology and intensive care medicine, outside the submitted work. Dr. Schaller holds stocks in small amounts from Alphabeth Inc., Bayer AG and Siemens AG; these holdings have not affected any decisions regarding his research or this study.

The authors declare that the research was conducted in the absence of any commercial or financial relationships that could be construed as a potential conflict of interest.

## Publisher’s note

All claims expressed in this article are solely those of the authors and do not necessarily represent those of their affiliated organizations, or those of the publisher, the editors and the reviewers. Any product that may be evaluated in this article, or claim that may be made by its manufacturer, is not guaranteed or endorsed by the publisher.
