# Supplementary material for: Successful treatment of out-of-hospital cardiac arrest is still based on quick activation of the chain of survival
Source: Front Public Health. 2023 Apr 11;11:1126503. doi: 10.3389/fpubh.2023.1126503 (PMC10126244; doi:10.3389/fpubh.2023.1126503)
Supplement: Supplementary file 1 [file Table_1.pdf]

Supplementary Material

Successful treatment of Out-of-hospital cardiac arrest is still based on quick activation of the chain of survival

Laura Borgstedt[1], Stefan J. Schaller[1,2], Daniel Goudkamp[1], Kristina Fuest[1], Bernhard Ulm[1], Bettina Jungwirth[3], Manfred Blobner[1,3], Sebastian Schmid[3]*

[1]Department of Anesthesiology and Intensive Care Medicine, School of Medicine, Technical University of Munich, Munich, Germany, [2]Department of Anesthesiology and Operative Intensive Care Medicine (CVK, CCM), Charité - Universitaetsmedizin Berlin, Berlin, Germany, [3]Department of Anesthesiology and Intensive Care, Faculty of Medicine, University of Ulm, Ulm, Germany

*** Correspondence:** Sebastian Schmid: s.schmid@uni-ulm.de

1 Supplementary Table 1.

Description of the entire cohort (n = 12,073 runs) which represents the total number of runs by the emergency physician staffed vehicle of the fire department 10 in Munich-Riem, Germany, from January 1st, 2014 – December 31st, 2017.

**Patients, n (%)**	
Men	5868 (48.6)
Women	5604 (46.4)
Sex unspecified/not documented	601 (5.0)
Age (mean (SD))	58.5 (24.9)
**Emergency location:**	
Home	7160 (59.3)
Other	2650 (21.9)
Public space	1396 (11.6)
School	89 (0.7)
Retirement home	778 (6.4)
**Major complaint:**	
Abdominal	797 (7.1)
Respiratory	923 (8.2)
Cardiovascular	3820 (34.0)
Not specified	1799 (16.0)
Central nervous system	1467 (13.1)
Other	923 (8.2)
Missing	846 (7.0)
Psychiatry	778 (6.9)
Metabolic	408 (3.6)
Pediatric	237 (2.1)
Gynecology	72 (0.6)
Neurology	3 (0.0)

2 Supplementary Files

2.1 Supplementary Image 1: Standardized EMS form DIVI version 4.2

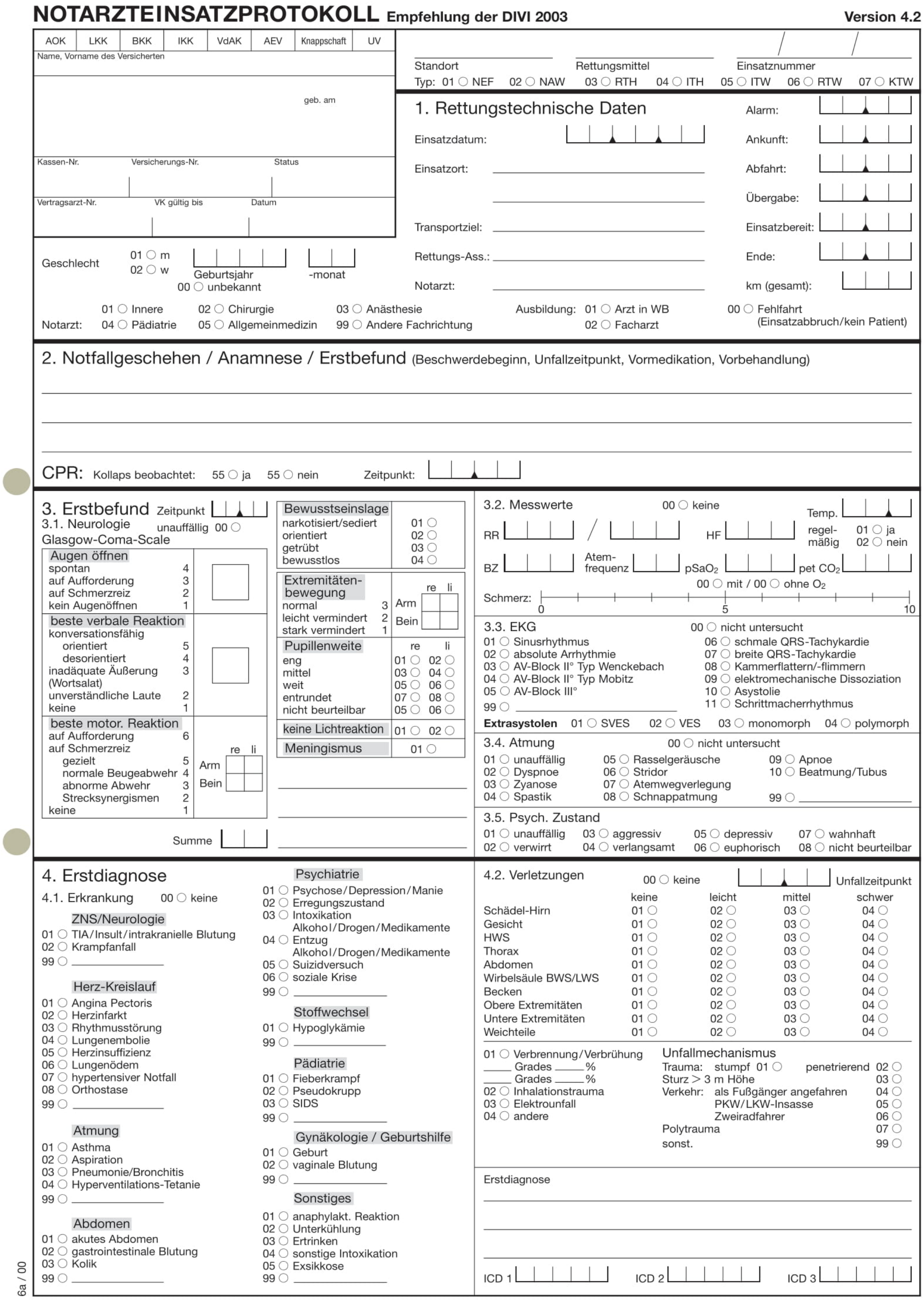

NOTARZTEINSATZPROTOKOLL Empfehlung der DIVI 2003 — Version 4.2

AOK | LKK | BKK | IKK | VdAK | AEV | Knappschaft | UV

Name, Vorname des Versicherten

geb. am

Kassen-Nr. | Versicherungs-Nr. | Status

Vertragsarzt-Nr. | VK gültig bis | Datum

Standort | Rettungsmittel | Einsatznummer

Typ: 01 ○ NEF 02 ○ NAW 03 ○ RTH 04 ○ ITH 05 ○ ITW 06 ○ RTW 07 ○ KTW

1. Rettungstechnische Daten

Einsatzdatum:

Einsatzort:

Transportziel:

Rettungs-Ass.:

Notarzt:

Alarm:

Ankunft:

Abfahrt:

Übergabe:

Einsatzbereit:

Ende:

km (gesamt):

Geschlecht 01 ○ m 02 ○ w

Geburtsjahr 00 ○ unbekannt

-monat

Notarzt: 01 ○ Innere 02 ○ Chirurgie 03 ○ Anästhesie 04 ○ Pädiatrie 05 ○ Allgemeinmedizin 99 ○ Andere Fachrichtung

Ausbildung: 01 ○ Arzt in WB 02 ○ Facharzt

00 ○ Fehlfahrt (Einsatzabbruch/kein Patient)

2. Notfallgeschehen / Anamnese / Erstbefund (Beschwerdebeginn, Unfallzeitpunkt, Vormedikation, Vorbehandlung)

CPR: Kollaps beobachtet: 55 ○ ja 55 ○ nein Zeitpunkt:

3. Erstbefund Zeitpunkt

3.1. Neurologie unauffällig 00 ○

Glasgow-Coma-Scale

Augen öffnen
spontan 4
auf Aufforderung 3
auf Schmerzreiz 2
kein Augenöffnen 1

beste verbale Reaktion
konversationsfähig
orientiert 5
desorientiert 4
inadäquate Äußerung (Wortsalat) 3
unverständliche Laute 2
keine 1

beste motor. Reaktion
auf Aufforderung 6
auf Schmerzreiz
gezielt 5
normale Beugeabwehr 4
abnorme Abwehr 3
Strecksynergismen 2
keine 1

re li — Arm — Bein

Summe

Bewusstseinslage
narkotisiert/sediert 01 ○
orientiert 02 ○
getrübt 03 ○
bewusstlos 04 ○

Extremitätenbewegung re li
normal 3
leicht vermindert 2
stark vermindert 1
Arm
Bein

Pupillenweite re li
eng 01 ○ 02 ○
mittel 03 ○ 04 ○
weit 05 ○ 06 ○
entrundet 07 ○ 08 ○
nicht beurteilbar 05 ○ 06 ○

keine Lichtreaktion 01 ○ 02 ○

Meningismus 01 ○

3.2. Messwerte 00 ○ keine

RR / HF Temp. regelmäßig 01 ○ ja 02 ○ nein

BZ Atemfrequenz $pSaO_2$ pet CO_2

00 ○ mit / 00 ○ ohne O_2

Schmerz: 0 5 10

3.3. EKG 00 ○ nicht untersucht
01 ○ Sinusrhythmus
02 ○ absolute Arrhythmie
03 ○ AV-Block II° Typ Wenckebach
04 ○ AV-Block II° Typ Mobitz
05 ○ AV-Block III°
99 ○
06 ○ schmale QRS-Tachykardie
07 ○ breite QRS-Tachykardie
08 ○ Kammerflattern/-flimmern
09 ○ elektromechanische Dissoziation
10 ○ Asystolie
11 ○ Schrittmacherrhythmus

Extrasystolen 01 ○ SVES 02 ○ VES 03 ○ monomorph 04 ○ polymorph

3.4. Atmung 00 ○ nicht untersucht
01 ○ unauffällig
02 ○ Dyspnoe
03 ○ Zyanose
04 ○ Spastik
05 ○ Rasselgeräusche
06 ○ Stridor
07 ○ Atemwegverlegung
08 ○ Schnappatmung
09 ○ Apnoe
10 ○ Beatmung/Tubus
99 ○

3.5. Psych. Zustand
01 ○ unauffällig 02 ○ verwirrt 03 ○ aggressiv 04 ○ verlangsamt 05 ○ depressiv 06 ○ euphorisch 07 ○ wahnhaft 08 ○ nicht beurteilbar

4. Erstdiagnose

4.1. Erkrankung 00 ○ keine

ZNS/Neurologie
01 ○ TIA/Insult/intrakranielle Blutung
02 ○ Krampfanfall
99 ○

Herz-Kreislauf
01 ○ Angina Pectoris
02 ○ Herzinfarkt
03 ○ Rhythmusstörung
04 ○ Lungenembolie
05 ○ Herzinsuffizienz
06 ○ Lungenödem
07 ○ hypertensiver Notfall
08 ○ Orthostase
99 ○

Atmung
01 ○ Asthma
02 ○ Aspiration
03 ○ Pneumonie/Bronchitis
04 ○ Hyperventilations-Tetanie
99 ○

Abdomen
01 ○ akutes Abdomen
02 ○ gastrointestinale Blutung
03 ○ Kolik
99 ○

Psychiatrie
01 ○ Psychose/Depression/Manie
02 ○ Erregungszustand
03 ○ Intoxikation Alkohol/Drogen/Medikamente
04 ○ Entzug Alkohol/Drogen/Medikamente
05 ○ Suizidversuch
06 ○ soziale Krise
99 ○

Stoffwechsel
01 ○ Hypoglykämie
99 ○

Pädiatrie
01 ○ Fieberkrampf
02 ○ Pseudokrupp
03 ○ SIDS
99 ○

Gynäkologie / Geburtshilfe
01 ○ Geburt
02 ○ vaginale Blutung
99 ○

Sonstiges
01 ○ anaphylakt. Reaktion
02 ○ Unterkühlung
03 ○ Ertrinken
04 ○ sonstige Intoxikation
05 ○ Exsikkose
99 ○

4.2. Verletzungen 00 ○ keine — Unfallzeitpunkt

	keine	leicht	mittel	schwer
Schädel-Hirn	01 ○	02 ○	03 ○	04 ○
Gesicht	01 ○	02 ○	03 ○	04 ○
HWS	01 ○	02 ○	03 ○	04 ○
Thorax	01 ○	02 ○	03 ○	04 ○
Abdomen	01 ○	02 ○	03 ○	04 ○
Wirbelsäule BWS/LWS	01 ○	02 ○	03 ○	04 ○
Becken	01 ○	02 ○	03 ○	04 ○
Obere Extremitäten	01 ○	02 ○	03 ○	04 ○
Untere Extremitäten	01 ○	02 ○	03 ○	04 ○
Weichteile	01 ○	02 ○	03 ○	04 ○

01 ○ Verbrennung/Verbrühung
____ Grades ____%
____ Grades ____%
02 ○ Inhalationstrauma
03 ○ Elektrounfall
04 ○ andere

Unfallmechanismus
Trauma: stumpf 01 ○ penetrierend 02 ○
Sturz > 3 m Höhe 03 ○
Verkehr: als Fußgänger angefahren 04 ○
PKW/LKW-Insasse 05 ○
Zweiradfahrer 06 ○
Polytrauma 07 ○
sonst. 99 ○

Erstdiagnose

ICD 1 ICD 2 ICD 3

6a / 00

5. Verlauf

h

Puls ○
RR
Defi
Intub.
HDM
Transport T-T

300
280
260
240
220
200
180
160
140
120
100
80
60
40

O_2 l/min
$pSaO_2$
pet CO_2
Maßnahmen

6. Maßnahmen

00 ○ keine

6.1. Herz/Kreislauf

01 ○ Herzdruckmassage
02 ○ Defibrillation/Kardioversion
03 ○ mono - biphasisch 09 ○
04 ○ biphasisch
05 ○ Erstdefibrillation durch RA/RS

Anzahl

1. Defi.

1. ROSC

Joule letzte Defi.

04 ○ peripher venöser Zugang — Anzahl

Ort/Größe

05 ○ zentral venöser Zugang — Anzahl

Ort/Größe

06 ○ intraossär. Zug.

07 ○ Schrittm. (ext.) 08 ○ Spritzenpumpe — Anzahl

6.2. Atmung

00 ○ keine

01 ○ Sauerstoffgabe — l/min
02 ○ Freimachen der Atemwege
03 ○ Absaugen
04 ○ Intubation — Größe Ch

05 ○ oral 06 ○ nasal
07 ○ LMA 08 ○ andere

09 ○ Beatmung 10 ○ manuell 11 ○ maschinell

AMV — AF
PEEP — FiO_2

6.3. Weitere Maßnahmen

00 ○ keine

01 ○ Narkose
02 ○ Blutstillung
03 ○ Magensonde
04 ○ Verband
05 ○ Reposition, Ort:
06 ○ besondere Lagerung, Art:
07 ○ Zervikalstütze
08 ○ Thoraxdrainage/Punktion
○ re ○ li Ch
Ort:
99 ○ Sonstiges

09 ○ Entbindung
10 ○ Dauerkatheter
11 ○ Krisenintervention
12 ○ Vakuummatratze

6.5 Medikamente

6.5 Medikamente	Dosis

00 ○ keine Medikamente
01 ○ nicht opiat Analgetika
02 ○ Opioide
03 ○ Antiarrhythmika
04 ○ Antidota
05 ○ Antiemetika
06 ○ Antiepileptika
07 ○ Antihypertensiva
08 ○ Bronchodilatantien
09 ○ Diuretika
10 ○ Glukose
11 ○ Katecholamine
12 ○ Kortikosteroide
13 ○ Muskelrelaxantien
14 ○ Narkotika
15 ○ Sedativa
16 ○ Thromb.aggr.hemmer
17 ○ Thrombolytikum
18 ○ Vasodilatantien
01 ○ Kristalloide
02 ○ Kolloide
03 ○ Pufferlösung
99 ○ Sonstige

6.4. Monitoring

00 ○ kein

01 ○ EKG-Monitor
02 ○ 12-Kanal-EKG
03 ○ Pulsoximetrie
04 ○ Kapnometrie
99 ○ Sonstiges
05 ○ manuelle Messung RR
06 ○ oszillometr. Messung RR
07 ○ Temperatur

7. Übergabe

7.1. Zustand

Bewusstseinslage
narkotisiert/sediert 01 ○
orientiert 02 ○
getrübt 03 ○
bewusstlos 04 ○

Glasgow-Coma-Scale

7.2. Messwerte

00 ○ keine

Temp.

RR / HF — regelmäßig 01 ○ ja 02 ○ nein

BZ — Atemfrequenz — $pSaO_2$ — et CO_2

Schmerz: 0 — 5 — 10

7.3. EKG

00 ○ nicht untersucht

01 ○ Sinusrhythmus
02 ○ absolute Arrhythmie
03 ○ AV-Block II° Typ Wenckebach
04 ○ AV-Block II° Typ Mobitz
05 ○ AV-Block III°
99 ○
06 ○ schmale QRS-Tachykardie
07 ○ breite QRS-Tachykardie
08 ○ Kammerflattern/-flimmern
09 ○ elektromechanische Dissoziation
10 ○ Asystolie
11 ○ Schrittmacherrhythmus

Extrasystolen 01 ○ SVES
02 ○ VES 03 ○ monomorph 04 ○ polymorph

7.4. Atmung

00 ○ nicht untersucht

01 ○ unauffällig
02 ○ Dyspnoe
03 ○ Zyanose
04 ○ Spastik
05 ○ Rasselgeräusche
06 ○ Stridor
07 ○ Atemwegsverlegung
08 ○ Schnappatmung
09 ○ Apnoe
10 ○ Beatmung/Tubus
99 ○

8. Ergebnis

8.1. Einsatzbeschreibung

01 ○ Transport ins Krankenhaus
02 ○ Sekundäreinsatz
03 ○ Patient lehnt Transport ab
04 ○ nur Untersuchung/Behandlung
05 ○ Übergabe an anderes Rettungsmittel
06 ○ Übernahme von arztbesetztem Rettungsmittel,
Art
07 ○ Reanimation primär erfolgreich
08 ○ Reanimation erfolglos
09 ○ Tod auf dem Transport
10 ○ Todesfeststellung
Zeit

8.2. Ersthelfermaßnahmen (Laien)

01 ○ suffizient
02 ○ insuffizent
03 ○ keine
04 ○ AED

8.3. Notfallkatetegorie

01 ○ kein Notfall
02 ○ akute Erkrankung
03 ○ Vergiftung
04 ○ Verletzung
Unfall
05 ○ Verkehr
06 ○ Arbeit
99 ○ Sonstiger

8.4. NACA-Score

01 ○ I geringfügige Störung
02 ○ II ambulante Abklärung
03 ○ III station. Behandlung
04 ○ IV akute Lebensgefahr nicht auszuschließen
05 ○ V akute Lebensgefahr
06 ○ VI Reanimation
07 ○ VII Tod

9. Bemerkung (z.B. Hausarzt)

Unterschrift Notarzt:

2.2 Supplementary Image 2: Standardized EMS form DIVI version 5.0

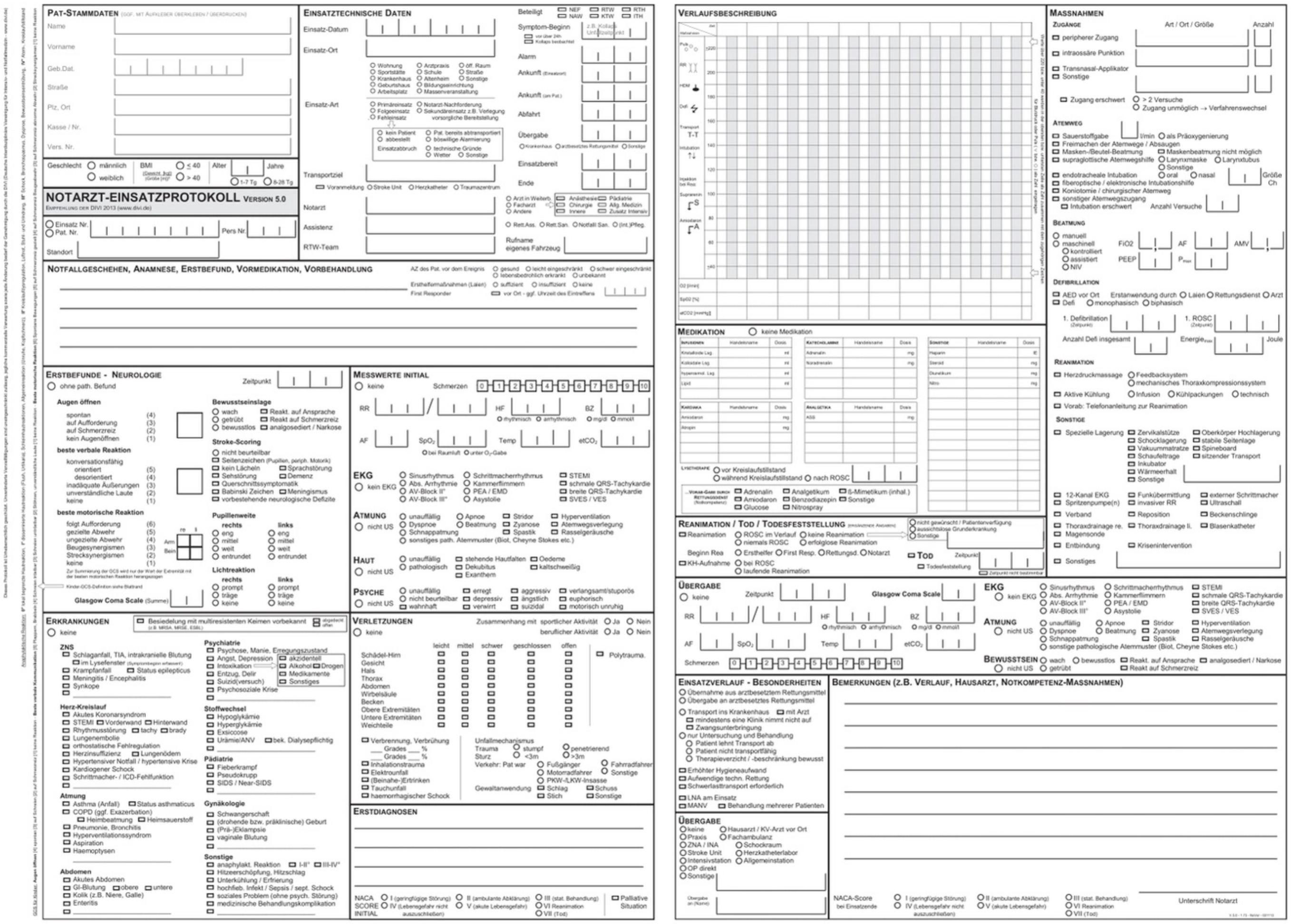

2.3 Supplementary Image 3: Standardized EMS form DIVI version 5.1

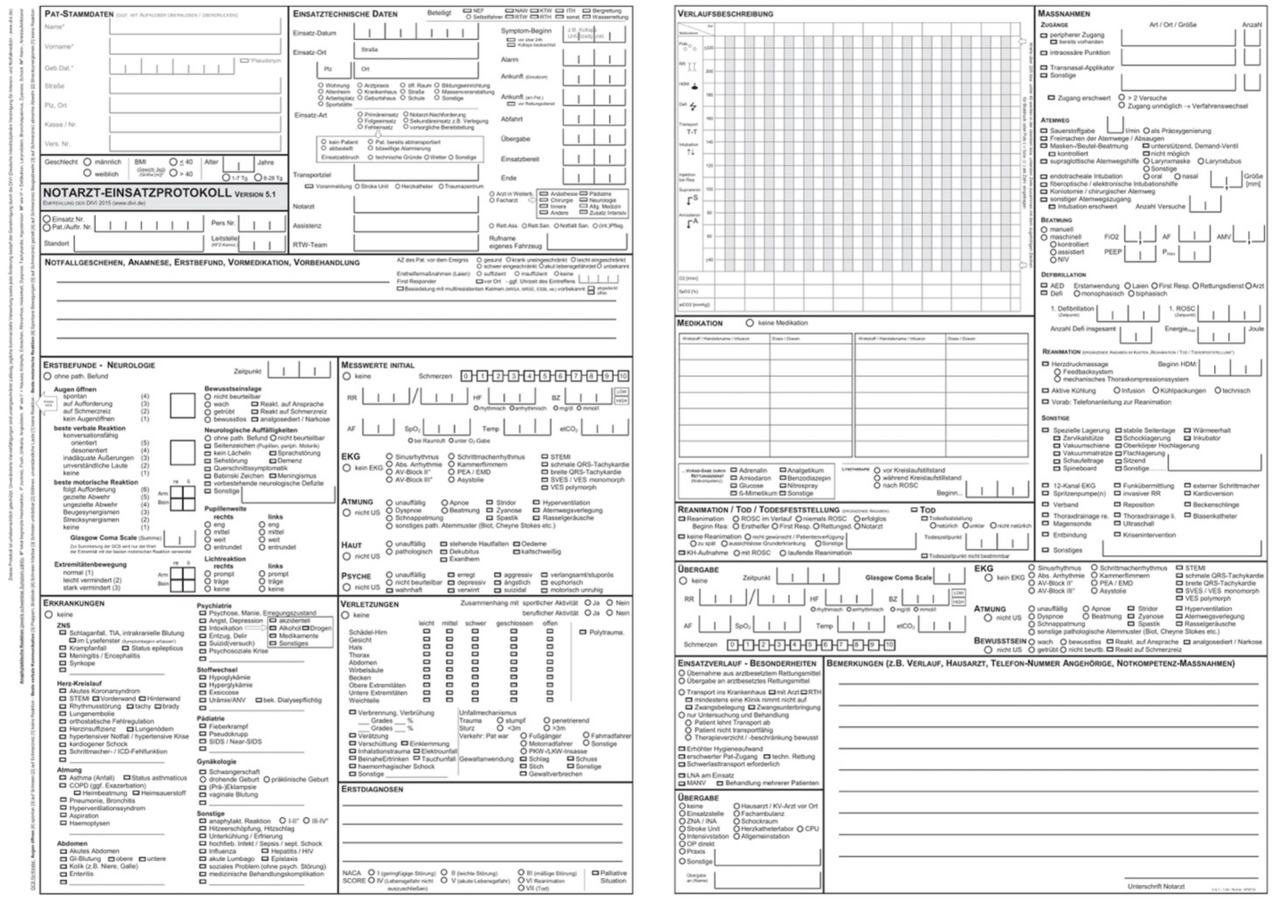
PAT-STAMMDATEN

EINSATZTECHNISCHE DATEN

NOTARZT-EINSATZPROTOKOLL VERSION 5.1

NOTFALLGESCHEHEN, ANAMNESE, ERSTBEFUND, VORMEDIKATION, VORBEHANDLUNG

ERSTBEFUNDE - NEUROLOGIE

MESSWERTE INITIAL

ERKRANKUNGEN

VERLETZUNGEN

ERSTDIAGNOSEN

VERLAUFSBESCHREIBUNG

MASSNAHMEN

MEDIKATION

REANIMATION / TOD / TODESFESTSTELLUNG

ÜBERGABE

EINSATZVERLAUF - BESONDERHEITEN

BEMERKUNGEN (Z.B. VERLAUF, HAUSARZT, TELEFON-NUMMER ANGEHÖRIGE, NOTKOMPETENZ-MASSNAHMEN)

ÜBERGABE

Unterschrift Notarzt
